# HbA1c at term delivery and adverse pregnancy outcome

**DOI:** 10.1186/s12884-022-05000-7

**Published:** 2022-09-03

**Authors:** Jesrine Gek Shan Hong, Mohd Yahaya Noor Fadzleeyanna, Siti Zawiah Omar, Peng Chiong Tan

**Affiliations:** Department of Obstetrics and Gynecology, Faculty of Medicine, Universiti Malaya, Jalan Profesor Diraja Ungku Aziz, 50603 Kuala Lumpur, Malaysia

**Keywords:** HbA1c, Glycated hemoglobin, Cesarean, Large for gestational age, Term, Adverse pregnancy outcome, Diabetes

## Abstract

**Background:**

There are no obvious thresholds at which the risk of adverse pregnancy outcomes increases as a result of maternal hyperglycemia. HbA1c level which is representative of average blood glucose levels over the last 2–3 months is not as strongly predictive of adverse outcome compared to blood glucose values of oral glucose tolerance test. Data is sparse on the predictive value of HbA1c at term just prior to delivery on adverse outcome. We sought to evaluate HbA1c taken on admission for delivery at term on adverse outcomes of Cesarean delivery and large (≥ 90^th^ centile) for gestational age (LGA) infants.

**Methods:**

A prospective cross-sectional study was conducted in a university hospital in Malaysia from December 2017-August 2018. 1000 women at term whose deliveries were imminent were enrolled. Blood were drawn and immediately sent for HbA1c analysis at our hospital laboratory. Primary outcomes were Cesarean delivery and LGA.

**Results:**

On crude analyses, Cesarean births (vs. vaginal births) were associated with significantly higher HbA1c (%) levels 5.4[5.2–5.7] vs. 5.3[5.1–5.6] *P* =  < 0.001 but not for LGA vs. non-LGA 5.4[5.1–5.6] vs. 5.3[5.1–5.6] *P* = 0.17. After controlling for significant confounders identified on crude analysis (diabetes in pregnancy, parity, ethnicity, body mass index (BMI), previous cesarean, labor induction, Group B streptococcus (GBS) carriage and birth weight), HbA1c is independently predictive of Cesarean birth, adjusted odds ratio (AOR) 1.47 95% CI 1.06–2.06 *P* = 0.023 per HbA1c 1% increase. Following adjustment for significant confounders (BMI, predelivery anemia [hemoglobin < 11 g/dl] and GBS carriage), the impact of raised HbA1c level on LGA is AOR 1.43 95% CI 0.93–2.18 *P* = 0.101 per HbA1c 1% increase and non-significant.

**Conclusion:**

Raised HbA1c level at term births in the general pregnant population is independently predictive of Cesarean delivery after adjustment for potential confounders including diabetes in pregnancy.

**Supplementary Information:**

The online version contains supplementary material available at 10.1186/s12884-022-05000-7.

## Background

Glycated hemoglobin (HbA1c) is used to assess long-term glycemic control in diabetes acting as a surrogate of glucose concentration in the preceding 8–12 weeks [1]. HbA1c “is a good predictor of diabetes-related complications” [2]. Of the HbA1c value, 50% is from glucose exposure in the last 30 days, 40% in the preceding 31–90 days and 10% in the previous 91–120 days [3]. HbA1c is convertible to estimated average blood glucose level at the ratio of 1% to 1.6 mmol/l [4] but in pregnancy 1% is reported to correspond to 0.67 mmol/l in average blood glucose [5]. HbA1c is slightly lower in normal pregnancy than in normal nonpregnant women [6] due in part to erythrocyte lifespan decrease from 120 to 90 days, and erythropoietin production increase during pregnancy [7].

The Hyperglycemia and Adverse Pregnancy Outcomes (HAPO) study reports that after 75-g oral glucose-tolerance testing (OGTT) at 24 to 32 weeks of gestation, there are no obvious thresholds at which risks increased and there exists a strong, continuous association of maternal glucose levels to adverse pregnancy outcomes. The impact of fasting, 1 and 2 h OGTT readings on adverse outcomes are comparable [8]. However, the HAPO study group also finds that “for each measure of fasting, 1-, and 2-h plasma glucose and A1C respectively higher by one standard deviation, odds ratios (ORs) for birth weight > 90th percentile were 1.39, 1.45, and 1.38 and 1.15 and for cord C-peptide > 90th percentile were 1.56, 1.45, and 1.35 and 1.32”. “ORs were similar for glucose and A1C for primary cesarean section, preeclampsia, and preterm delivery” and concludes that “A1C measurement is not a useful alternative to an OGTT in pregnant women” [2]. Hence ‘real time’ OGTT blood glucose values appear to better reflect the dynamic glycemic status as pregnancy goes forward compared to HbA1c which represents retrospectively the average blood glucose over the preceding 8–10 weeks of the pregnancy.

HbA1c level at delivery or term reflecting cumulative glycemic history of the preceding two to three months may have the potential to be a surrogate measure for adverse pregnancy outcome as theoretically supported by HAPO study observation that there were no obvious glycemic thresholds at which risks increased [8]. We hypothesized that raised HbA1c at term just prior to delivery will be predictive of Cesarean birth and LGA.

## Methods

### Participants

Women were assessed for study eligibility by scrutinizing their medical records when they were admitted for delivery to the antenatal and labor wards of the Obstetrics Unit. Inclusion criteria were women presenting for imminent delivery (in spontaneous labor, scheduled induction of labor or planned cesarean) at our labor or antenatal wards who were aged ≥ 18 years, with a singleton pregnancy and at term gestation (≥ 37 weeks confirmed by ultrasound before 22 weeks gestation). We excluded women with severe-moderate to severe anemia in pregnancy (hemoglobin level < 8 g/dl) [9, 10], known major hemoglobinopathy [11, 12], known gross fetal anomaly (as these characteristics might have major impact on HbA1c assessment or birth weight) and inability to consent due to language difficulty.

Eligible women were approached, provided with the Patient Information Sheet and had oral queries answered by the recruiting investigator (co-author MYNF). Written informed consent to participate in the study was obtained from all participants. All participants’ relevant characteristics including diagnosis of prepregnant diabetes mellitus or gestational diabetes mellitus (GDM), current use of antiglycemic agents, hypertension in pregnancy, positive Group B streptococcus culture during pregnancy, obstetric history e.g., previous Cesarean and parity were transcribed onto the Case Report Form.

### Recruitment and interventions

Women who planned delivery at our center were routinely screened for gestational diabetes with the 75-g OGTT (based on Malaysian GDM screening criteria and diagnostic thresholds: fasting ≥ 5.1 and/or 2-h ≥ 7.8 mmol/l) [13] at booking and/or 24–28 weeks gestation depending on risk factors, hepatitis B, HIV infection, and had dating ultrasound in early pregnancy. Women with diabetes in pregnancy were monitored by their blood sugar profiles through self-monitoring of blood glucose. In women with diabetes in pregnancy, delivery (usually by labor induction unless contraindicated) is arranged by no later than 40 weeks gestation or earlier if any concerning clinical factors were present. Women who delivered at our center usually had a full blood count taken at their birth admission amongst other indicated blood tests if any.

Three milliliters of venous blood were drawn from participants, typically piggy-backed to venipuncture for routine bloods or at insertion of an indwelling intravenous catheter for delivery according to our care protocol. The blood was placed in a EDTA (ethylenediaminetetraacetic acid) blood bottle and dispatched to our hospital laboratory for immediate processing to establish HbA1c level. Our laboratory utilized Biorad Variant 2, Chemopharm, Selangor, Malaysia to run the blood samples using high performance liquid chromatography.

The predelivery HbA1c results were not revealed to participants and care providers.

### Outcome measures

Primary outcomes were Cesarean delivery and LGA (≥ 90 centile for gestational age birth weight), which were two of the four primary adverse primary outcomes of the original 2008 HAPO study [8] and that are also used in the setting of GDM diagnostic thresholds by IADPSG in 2010 [14].

### Sample size calculation

For sample size calculation the following principles were considered: “for regression equations using six or more predictors, an absolute minimum of 10 participants per predictor variable is appropriate” [15] and “a minimum of 10 cases with the least frequent outcome for each independent variable in your model” [16].

We assumed 10 independent variables in the model and probability of the least frequent outcome is 0.10, hence the sample size calculated is 10 × 10 / 0.10 = 1000. In our center, the Cesarean delivery rate was about 30%, hence a sample size of 1000 was expected to yield 300 Cesarean events and with LGA defined as birth weight ≥ 90^th^ centile corrected for gestational age, sample size of 1000 should yield 100 LGA events. Both these number of event estimates should permit a robust binary logistic regression analysis model of up to 10 independent covariables whilst keeping to the 10 event per variable rule.

### Statistical analyses

Data were entered into a statistical software package SPSS (Version 23, IBM, SPSS Statistics). The collected participant characteristics were analyzed against the primary outcomes of Cesarean vs. non-Cesarean delivery and LGA vs. non-LGA separately. Birth weight was included as a surrogate for estimated fetal weight [17] in the model for Cesarean delivery. The t test was used to compare means of continuous data, Mann–Whitney U test for non-parametric data and Chi-square test to analyze categorical data to yield crude results. In adjusted analysis, independent co-variables (identified confounders) with *p* < 0.05 on crude analysis were incorporated into the multivariable binary logistic regression model to control for confounders on the impact of HbA1c level on the primary outcomes. Post hoc adjusted analyses on different subsets of our study population and also for other adverse outcome (postpartum hemorrhage) were performed incorporating HbA1c level and all the initially identified confounders. Two-sided *P* values were reported and *P* < 0.05 was considered as significant.

## Results

**Fig. 1:**
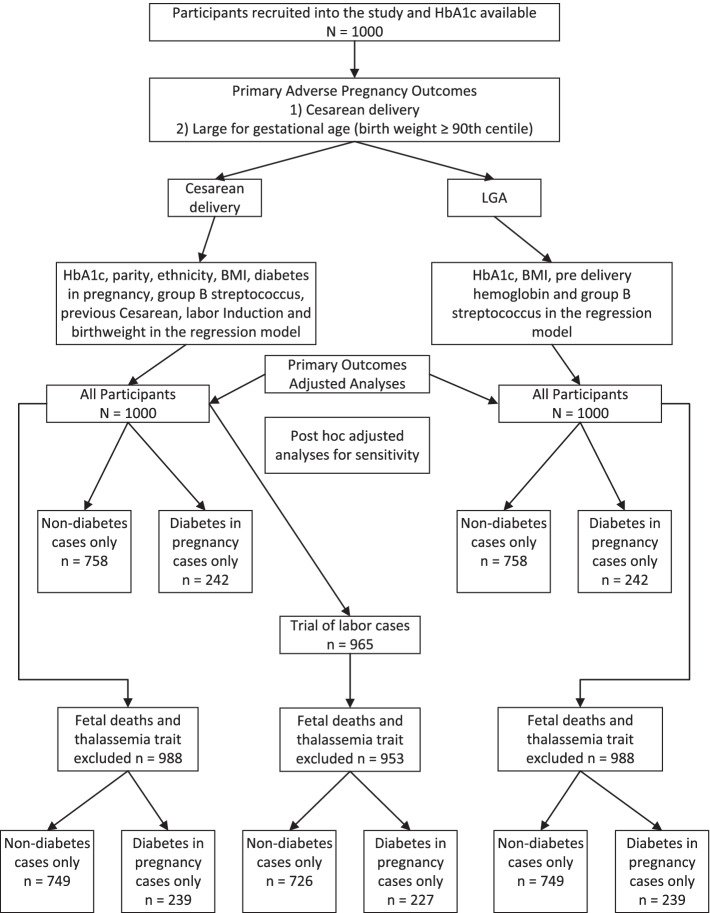
Study flow chart of HbA1c as predictor of adverse pregnancy outcomes and adjusted analyses models

We recruited 1000 eligible women just prior to their delivery at term in our medical center. Venous blood samples were sent to our laboratory for HbA1c analysis and results were available. The enrolment and adjusted analyses flow are shown on Fig. 1.

### Participants’ characteristics and pregnancy outcomes

Table 1 lists the characteristics of the study participants. The median [interquartile range] of HbA1c of the study women was 5.3 [5.1–5.6] %. 242/1000 (24.2%) had diabetes in pregnancy, predominantly GDM (232/242—95.9%). 70/242 (28.9%) of these women with diabetes in pregnancy were on antiglycemic drug treatment. The primary outcomes Cesarean rate was 307/1000 (30.7%), 272/307 (88.6%) were unplanned following a failed trial of labor or induction of labor and LGA rate is 99 (9.9%), both incidences in line with estimates used in the sample size calculation. There were two cases of fetal deaths at presentation.

**Table 1:** Characteristics and outcomes of study population

Characteristics	Participants (*N* = 1000)
**HbA1c (%)**	5.3 [5.1–5.6]
HbA1c ≤ 5.6%	794 (79.4%)
HbA1c 5.7–6.4%	197(19.7%)
HbA1c ≥ 6.5%	9 (0.9%)
**Gestational age (weeks, mean ± SD)**	39.0 ± 1.1
**Age (years, mean ± SD)**	30.6 ± 4.6
**Parity (median [IQR])**	1 [0–2]
0	396 (39.6%)
1	325 (32.5%)
2	166 (16.6%)
≥ 3	113 (11.3%)
**Previous miscarriage**	212 (21.2%)
**Ethnicity**	
Malay	621 (62.1%)
Chinese	127 (12.7%)
Indian	116 (11.6%)
Others	136 (13.6%)
**BMI (kg/m**^**2**^**, mean ± SD)**	28.9 ± 4.9
< 25	215 (21.5%)
25–29.9	431 (43.1%)
≥ 30	354 (35.4%)
**Hemoglobin, predelivery (g/dL, mean ± SD)**	12.0 ± 1.3
**Medical & Obstetric History**	
**Diabetes in pregnancy**	242 (24.2%)
Gestational diabetes	232 (23.2%)
Type 2 diabetes	10 (1.0%)
**Antiglycemic drug in pregnancy**	70 (7.0%)
Metformin only	54 (5.4%)
Insulin only	3 (0.3%)
Metformin and insulin	13 (1.3%)
**Asthma**	53 (5.3%)
**Hypertension in pregnancy**	49 (4.9%)
**Group B streptococcus carriage**	59 (5.9%)
**Thalassemia trait**	10 (1.0%)
**HIV or Hepatitis B infection**	9 (0.9%)
**Previous Caesarean**	165 (16.5%)
**Induction of labor**	206 (20.6%)
**Outcomes**	
**Caesarean delivery**	307 (30.7%)
Planned	35 (3.5%)
Unplanned	272 (27.2%)
**Birth weight**	3.08 ± 0.40
LGA (Large for gestational age)	99 (9.9%)
LBW (low birth weight < 2.5 kg)	66 (6.6%)
**Estimated delivery blood loss**	323 ± 247
PPH ≥ 500 ml	146 (14.6%)
**Neonatal admission (*n* = 998)**	54 (5.4%)
**Cord artery blood pH (*n* = 979)**	7.30 ± 0.07
pH < 7.1	15 (1.5%)
**Cord artery blood base excess (*n* = 969)**	-4.03 ± 3.41
BE ≤ -12	24 (2.4%)
**Apgar score at 1 min (median [IQR]) (*n* = 998)**	9 [9-9]
Apgar score at 1 min ≤ 3	2 (0.2%)
**Apgar score at 5 min (median [IQR]) (*n* = 998)**	10 [10-10]
Apgar score at 5 min ≤ 6	1 (0.1%)

Values are stated as mean ± standard deviation, median [interquartile range] or n (%). Analysis by t test for continuous data, Mann–Whitney U test for non-parametric data and Chi Square test for categoric variable

### Crude and adjusted analyses

Table 2 depicts the analysis on the crude effect of independent covariables on Cesarean delivery and the adjusted analysis. On crude analysis, Cesarean delivery was significantly (*p* < 0.05) associated with HbA1c, parity, ethnicity, body mass index (BMI), diabetes in pregnancy, Group B streptococcus ano-vaginal positive culture, previous Cesarean, induction of labor and birthweight. On adjusted analysis controlling for these confounders, Cesarean delivery was still significantly associated with raised HbA1c, AOR (adjusted odds ratio) 1.47 95% CI (Confidence Interval) 1.06–2.06 *P* = 0.023 for each 1% increase in HbA1c or alternatively stated AOR (adjusted odds ratio) 1.04 95% CI (Confidence Interval) 1.01–1.08 *P* = 0.023 for each 0.1% increase in HbA1c.

**Table 2:** Characteristics (independent variables) dichotomized according to Cesarean delivery and vaginal delivery (dependent variable) on bivariate or crude analysis and results following adjusted analysis incorporating all significant (*p* < 0.05) independent variables on crude analysis

Variable	Cesarean Delivery (*n* = 307)	Vaginal Delivery (*n* = 693)	*P* Value	RR (95% CI)	Multivariable Logistic Regression Analysis	
					AOR (95%CI)	*P* value
**HbA1c (%)**	5.4[5.2–5.7]	5.3[5.1–5.6]	< 0.001		1.47 (1.06–2.06)^a^	0.023
					1.04 (1.01–1.08)^b^	0.023
**Gestational age (weeks) mean ± SD)**	39.1 ± 1.2	39.0 ± 1.1	0.21			
**Age (years, mean ± SD)**	30.7 ± 4.4	30.6 ± 4.7	0.64			
**Parity category**			0.001			< 0.001
0	148 (48.2%)	248 (35.8%)				
1	92 (30.0%)	233 (33.6%)			0.29 (0.20–0.43)	< 0.001
2	44 (14.3%)	122 (17.6%)			0.27 (0.17–0.45)	< 0.001
≥ 3	23 (7.5%)	90 (13.0%)			0.19 (0.10–0.34)	< 0.001
**Previous miscarriage**	63 (20.5%)	149 (21.5%)	0.73	0.95 (0.73–1.24)		
**Ethnicity**			< 0.001			0.006
Malay	169 (55.0%)	452 (65.2%)				
Chinese	37 (12.1%)	90 (13.0%)			1.17 (0.73–1.87)	0.51
Indian	55 (17.9%)	61 (8.8%)			2.00 (1.28–3.12)	0.002
Other	46 (15.0%)	90 (13.0%)			1.71 (1.10–2.68)	0.018
**BMI (kg/m**^**2**^**, mean ± SD)**	30.0 ± 5.0	28.4 ± 4.7	< 0.001		1.06 (1.02–1.09)	0.001
Hemoglobin < 11 g/dl	74 (24.1%)	138 (19.9%)	0.14	1.28 (0.93–1.76)		
**Medical & Obstetric history**						
**Diabetes in pregnancy**	88 (28.7%)	154 (22.2%)	0.028	1.29 (1.03–1.62)	1.07 (0.75–1.53)	0.72
Anti-glycemic	24 (7.8%)	46 (6.6%)	0.50	1.18 (0.73–1.89)		
**Asthma**	15 (4.9%)	38 (5.5%)	0.70	0.89 (0.50–1.60) 1.591.59)(0.501.5911.591.59)_		
**Hypertension in pregnancy ppregnncypregnancy**	21(6.8%) (6.8%)	28 (4.0%)	0.059	1.69 (0.98–2.93)		
**Group B streptococcus carriage**	25 (8.1%)	34 (4.9%)	0.045	1.66 (1.01–2.73)	1.83 (1.01–3.33)	0.049
**Thalassemia trait**	3 (1.0%)	7 (1.0%)	0.96	0.97 (0.25–3.72)		
**HIV or Hepatitis B infection**	0 (0.0%)	9 (1.3%)	0.064	*		
**Previous Cesarean**	93 (30.3%)	72 (10.4%)	< 0.001	2.92 (2.21–3.85)	7.30 (4.76–11.17)	p < 0.001
**Induction of labor**	86 (28.0%)	120 (17.3)	< 0.001	1.62 (1.27–2.06)	1.85 (1.28–2.67)	0.001
**Birth weight (kg)**	3.14 ± 0.41	3.06 ± 0.39	0.003		1.82 (1.23–2.70)	0.003

Values are stated as mean ± standard deviation, median [interquartile range] or n (%). Crude analysis by t test for continuous data, Mann–Whitney U test for non-parametric data, and Chi Square test for categoric variable. Adjustment made utilizing multivariable binary logistic regression analysis of significant independent variable with adjusted results shown if variable incorporated in the model

^a^ per 1% increase in HbA1c level

^b^ per 0.1% increase in HbA1c level

Table 3 shows the analysis on the crude effect of independent covariables on LGA. On crude analysis, LGA was significantly associated with BMI, predelivery anemia (hemoglobin < 11 g/dl) [9] and Group B streptococcus carriage. On adjusted analysis controlling for these confounders, LGA was not significantly associated with raised HbA1c, AOR 1.43 95% CI 0.93–2.18 *P* = 0.101 for each 1% increase in HbA1c.

**Table 3:** Characteristics (independent variables) dichotomized according to Cesarean delivery and vaginal delivery (dependent variable) on bivariate or crude analysis and results following adjusted analysis incorporating all significant (p < 0.05) independent variables on crude analysis

Variable	LGA (*n* = 99)	Non LGA (*n* = 901)	*P* Value	RR (95% CI)	Multivariable Logistic Regression Analysis	
					AOR (95%CI)	*P* value
**HbA1c (%)**	5.4[5.1–5.6]	5.3[5.1–5.6]	0.17		1.43 (0.93–2.18)	0.101
**Gestational age (weeks, mean ± SD)**	39.1 ± 1.1	39.0 ± 1.1	0.55			
**Age (years, mean ± SD)**	31.0 ± 4.5	30.6 ± 4.6	0.43			
**Parity category**			0.068			
0	29 (29.3%)	367 (40.7%)				
1	37 (37.4%)	288 (32.0%)				
2	16 (16.2%)	150 (16.6%)				
≥ 3	17 (17.2%)	96 (10.7%)				
**Previous miscarriage**	23 (23.2%)	189 (21.0%)	0.60	1.11 (0.76–1.62)		
**Ethnicity**			0.81			
Malay	66 (66.7%)	555 (61.6%)				
Chinese	11 (11.1%)	116 (12.9%)				
Indian	10 (10.1%)	106 (11.8%)				
Others	12 (12 .1%)	124 (13.8%)				
**BMI (kg/m**^**2**^**, mean ± SD)**	29.9 ± 4.8	28.8 ± 4.9	0.022		1.04 (1.00–1.09)	0.048
**Hemoglobin < 11 g/dL**	30 (30.3%)	182 (20.2%)	0.02	1.50 (1.08–2.08)	1.74 (1.09–2.78)	0.021
**Medical and obstetric history**						
**Diabetes in pregnancy**	23 (23.2%)	219 (24.3%)	0.81	0.96 (0.66–1.39)		
Antiglycemic	7 (7.1%)	63 (7.0%)	0.98	1.01 (0.48–2.15)		
**Asthma**	5 (5.1%)	48 (5.3%)	0.91	0.95 (0.39–2.33)		
**Hypertension in pregnancy**	6 (6.1%)	43 (4.8%)	0.57	1.27 (0.56–2.91)		
**Group B streptococcus carriage**	12 (12.1%)	47 (5.2%)	0.006	2.32 (1.28–4.23)	2.71 (1.37–5.38)	0.004
**Thalassemia trait**	0 (0.0%)	10 (1.1%)	0.29	*		
**HIV or Hepatitis B infection**	1 (1.0%)	8 (0.9%)	0.61	1.14 (0.14–9.00)		
**Previous Cesarean**	17 (17.2%)	148 (16.4%)	0.85	1.05 (0.66–1.65)		
**Induction of labor**	16 (16.2%)	190 (21.1%)	0.25	0.77 (0.48–1.17)		

Values are stated as mean ± standard deviation, median [interquartile range] or n (%). Crude analysis by t test for continuous data, Mann–Whitney U test for non-parametric data and Chi Square test for categoric variable, Adjustment made utilizing multivariable binary logistic regression analysis of significant independent variable with adjusted results shown if variable incorporated in the model

### Post hoc analyses

Post-hoc sensitivity analysis to evaluate the adjusted impact per 1% increase in HbA1c for risk of Cesarean delivery shows AOR 1.90 95% CI 1.24–2.91 *P* = 0.003 (pregnancies unaffected by diabetes) and AOR 0.84 95% CI 0.45–1.59 *P* = 0.600 (diabetes in pregnancy) (Supplementary Table S1). For risk of LGA, AOR 1.11 95% CI 0.64–1.94 *P* = 0.703 (pregnancies unaffected by diabetes) and AOR 2.35 95% CI 1.10–5.03 *P* = 0.027 (diabetes in pregnancy) per 1% increase in HbA1c. Adjusted analysis after excluding cases affected by fetal death (2 cases) which can confound results for Cesarean delivery and LGA and thalassemia trait (10 cases) which can confound on reliability of HbA1c assay [18] did not materially affect results. Similarly, adjusted analysis restricted to cases after a trial of labor or induction of labor that excluded the 35 cases of planned Cesareans also did not materially affect results.

On risk of Cesarean delivery, adjusted analysis separately of nondiabetic and diabetes in pregnancy participants demonstrated an unexpected change in the directionality of the point estimate of impact of HbA1c although the result is not significant, showing a major attenuation in the impact of raised HbA1c for diabetes in pregnancy cases and enhancement in nondiabetics. On the other hand, with regard to risk of LGA, the directionality of the point estimate of impact of HbA1c was similar for non-diabetic and diabetes in pregnancy participants, the impact was attenuated in non-diabetics and enhanced in diabetes in pregnancy cases (Supplementary Table S1).

We also looked at adverse pregnancy outcome of postpartum hemorrhage (estimated peri delivery loss ≥ 500 ml) as there was a significant number of such cases (*n* = 165) for regression analysis. On crude analysis (*N* = 1000) PPH cases had higher HbA1c (%) 5.4 [5.2–5.6] vs. 5.3 [5.1–5.6] *P* = 0.033 compared to non-PPH cases. After adjusted analysis controlling for significant confounders of BMI, predelivery hemoglobin, diabetes in pregnancy, previous Cesarean, infant birth weight and current delivery by Cesarean, the impact of HbA1c was significantly attenuated AOR 1.20 95% CI 0.78–1.82 *P* = 0.41 per 1% increase in HbA1c (Supplementary Table S2).

## Discussion

Predelivery HbA1c at term in the general pregnant population is a potential predictor for adverse pregnancy outcomes. HbA1c might be a useful integrated marker for studies assessing impact of interventions to control hyperglycemia through pregnancy.

In our prospective study of the general pregnant population at term just prior to delivery, maternal HbA1c was raised in Cesareans and but not LGA infants’ cases compared to relevant controls on crude analysis. There was a similar association on post hoc analysis with PPH. Following adjusted analysis to control for confounders, HbA1c was independently predictive of Cesarean delivery. Its impact on LGA was not improved and on PPH was especially attenuated after adjustment and was no longer significant. Various sensitivity analyses confirmed HbA1c as independently predictive of Cesarean delivery. Raised HbA1c appeared to have a greater impact on Cesarean delivery in women unaffected by diabetes in pregnancy than in affected pregnancies, whereas it appeared to have a greater impact on LGA in women affected by diabetes in pregnancy than in the unaffected women.

HbA1c ≥ 5.9% measured at a median 47 days' gestation at antenatal booking in the general pregnant population predicts early GDM, major congenital anomaly, preeclampsia, shoulder dystocia and perinatal death [19]. HAPO data shows that HbA1c has comparable predictive power to the various glucose values of the 75-g OGTT when both were contemporaneously obtained at 24 to 36 weeks gestation with regard to primary Cesarean delivery [2]. Our finding of positive impact on Cesarean by HbA1c at term just prior to delivery corroborated HAPO findings at late second to early third trimesters, also in the general pregnant population without obvious diabetes.

HAPO data also shows that HbA1c has somewhat weaker predictive value to the blood glucose values of the 75-g OGTT with regard to birth weight > 90^th^ centile [2]. A large prospective nationwide birth cohort study reports that “the higher the HbA1c level (before 24 weeks gestation), the higher the risk of adverse pregnancy outcomes in Japan” including on LGA [20]. In a multiethnic population, an early HbA1c ≥ 5.9% identifies high risk for macrosomia independently of GDM [21]. Our finding that the predictive value of HbA1c on LGA was still not significant after adjustment for potential confounders was in good keeping on the performance of HbA1c within HAPO on the adverse pregnancy outcome metric of a large baby. It is commented that “as A1C represents an integrated measure of glucose, it may not fully capture postprandial hyperglycemia, which drives macrosomia” [6] which could have explained our finding on attenuated LGA risk. It is plausible that our finding on LGA after adjustment is a Type 2 error due to inadequate sample size as LGA cases were only 99/1000.

A higher HbA1c level within the normal range is an independent risk factor for preterm delivery and preeclampsia, especially among GDM-negative women” [22]. In our post hoc analysis with a different adverse pregnancy outcome metric of unplanned Cesarean delivery, we find that predelivery HbA1c at term was a stronger predictor in women unaffected by diabetes in pregnancy than in affected women. Caution is needed as our subgroup analysis in women affected by diabetes in pregnancy comprised only 227 women with 76 Cesarean deliveries.

There is a lack of reliable predictive tools for adverse pregnancy outcomes in pregnancies complicated by diabetes. As a predictor variable, the pulsatility index of the umbilical artery from Doppler assessment shows an inversely related to birthweight centile [23] and to LGA in pregnancies affected by hyperglycaemia [24].

HbA1c just prior to delivery at term has the potential to be a useful integrated marker for adverse pregnancy outcomes in a pregnant population comprising those with identified diabetes in pregnancy and those previously screened negative for GDM. The study timing for HbA1c was obviously too advanced into pregnancy to help management to minimize the impact of hyperglycemia on adverse pregnancy outcome. It could still be clinically useful to guide care in women at high risk of Cesarean delivery, for instance in labor induction with other adverse factors for a failed induction or considering a trial of labor with previous Cesarean. HbA1c at term also has the potential as an integrated marker and predictor for adverse pregnancy outcome in studies assessing interventions to ameliorate the impact of hyperglycemia through pregnancy. Further study in a wider range of settings is needed to corroborate our findings.

### Strengths and limitations

As to strengths, our study is original in the prospective evaluation of just prior to delivery HbA1c at term on adverse outcomes that systemically controlled for identified confounders in a general pregnant population. Our population was well defined and our sample size appropriately calculated for a multivariable binary logistic regression analysis. The observed frequencies of our two primary outcomes were as assumed. Our study population was multiethnic Asian and we believed our results would be generalizable to a similar population and care setting.

As to limitations, this study was not appropriately powered to assess the impact of HbA1c especially in the smaller subgroup of women with diabetes. There was suggestion from our data that in women with diabetes, HbA1c is less powerful in predicting Cesarean delivery but more powerful with regard to predicting LGA. The underlying reasons were not clear but there might be confounding interactions arising from subjective decision making on planned, and unplanned Cesareans during a trial of labor once cases were recognized to be affected by diabetes. On the other hand, on the more objective adverse outcome of LGA, the point estimate directionality of raised HbA1c was in the same direction albeit with greater impact amongst women with diabetes in pregnancy than those without.

## Conclusion

Raised HbA1c just prior to a term delivery in a general pregnant population correlate positively with Cesarean delivery and postpartum hemorrhage. After adjustment for identified confounders, only Cesarean delivery risk is independently associated with increased HbA1c levels.

## Supplementary Information

**Additional file 1.**

**Additional file 2.**

## Data Availability

The datasets used and/or analyzed during the current study are available from the corresponding author on reasonable request.

## Abbreviations

BMI: Body mass index
GDM: Gestational diabetes mellitus
HbA1c: Glycated hemoglobin
LGA: Large for gestational age
OGTT: Oral glucose tolerance test
PPH: Postpartum hemorrhage
