# Supplementary material for: HbA1c at term delivery and adverse pregnancy outcome
Source: BMC Pregnancy Childbirth. 2022 Sep 3;22:679. doi: 10.1186/s12884-022-05000-7 (PMC9440566; doi:10.1186/s12884-022-05000-7)
Supplement: Supplementary file 1 — Additional file 1. [file 12884_2022_5000_MOESM1_ESM.docx]

**Supplementary Table S1.** Post hoc analysis. Adjusted odd ratio (95% CI) P value for HbA1c after adjustment on outcomes of Cesarean delivery and large for gestational age

| All Cesarean delivery (N = 1000) | | Large for gestational (N = 1000) | |
| --- | --- | --- | --- |
|  | AOR (95% CI) P value |  | AOR (95% CI) P value |
| Non diabetes only^3^  (n = 758) | 1.90 (1.24-2.91) 0.003 | Non diabetes only^3^  (n = 758) | 1.11 (0.64-1.94) 0.703 |
|  |  |  |  |
| Diabetes only^4^  (n = 242) | 0.84 (0.45-1.59) 0.600 | Diabetes only^4^  (n = 242) | 2.35 (1.10-5.03) 0.027 |
|  |  |  |  |
| Fetal death or thalassemia trait excluded  (n = 988) | 1.50 (1.08-2.10) 0.017^1^ | Fetal death or thalassemia trait excluded  (n = 988) | 1.43 (0.94-21.9) 0.096^2^ |
|  |  |  |  |
| Non diabetes only^3^  Fetal death or thalassemia trait excluded (n = 749) | 1.92 (1.25-2.96) 0.003^1^ | Non diabetes only^3^  Fetal death or thalassemia trait excluded (n = 749) | 1.12 (0.64-1.94) 0.699^2^ |
|  |  |  |  |
| Diabetes only^4^  Fetal death or thalassemia trait excluded (n = 239) | 0.89 (0.47-1.67) 0.712^1^ | Diabetes only^4^  Fetal death or thalassemia trait excluded (n = 239) | 2.38 (1.11-5.10) 0.026^2^ |
|  |  |  |  |
|  |  |  |  |
| Unplanned Cesarean delivery (n=965) | |  |  |
|  | AOR (95% CI) P value |  |  |
| Unplanned Cesarean (n=965) | 1.42 (1.01-2.00) 0.044^1^ |  |  |
|  |  |  |  |
| Fetal death or thalassemia trait excluded (n = 953) | 1.45 (1.03-2.04) 0.035^1^ |  |  |
|  |  |  |  |
| Non diabetes only^3^  Fetal death or thalassemia trait excluded (n = 726) | 1.86 (1.20-2.90) 0.006^1^ |  |  |
|  |  |  |  |
| Diabetes only^4^  Fetal death or thalassemia trait excluded (n = 227) | 0.81 (0.41-1.61) 0.553^1^ |  |  |
|  |  |  |  |

^1^Regression model includes as independent covariables HbA1c, parity, ethnicity, body mass index, diabetes in pregnancy, group B streptococcus carriage, previous Cesarean, labor induction and birth weight

^2^Regression model includes as independent covariables HbA1c, body mass index, predelivery hemoglobin < 11 g/dl and group B streptococcus carriage

^3^Non diabetes in pregnancy cases after gestational diabetes screening

^4^Gestaional diabetes or Type 2 diabetes
