# Supplementary material for: HbA1c at term delivery and adverse pregnancy outcome
Source: BMC Pregnancy Childbirth. 2022 Sep 3;22:679. doi: 10.1186/s12884-022-05000-7 (PMC9440566; doi:10.1186/s12884-022-05000-7)
Supplement: Supplementary file 2 — Additional file 2. [file 12884_2022_5000_MOESM2_ESM.docx]

**Supplementary Table S2.** Characteristics (independent variables) dichotomized according to PPH (postpartum hemorrhage ≥ 500 ml) and no PPH (dependent variable) on bivariate or crude analysis and results following adjusted analysis incorporating all significant (p < 0.05) independent variables on crude analysis.

| Variable | PPH (n=146) | No PPH (n=854) | P Value | RR (95% CI) | Multivariable Logistic Regression Analysis | |
| --- | --- | --- | --- | --- | --- | --- |
|  |  |  |  |  | AOR (95%CI) | P value |
| **HbA1c (%)** | 5.4[5.2-5.6] | 5.3[5.1-5.6] | 0.033 |  | 1.20 (0.78-1.82) | 0.41 |
| **Gestational age (weeks, mean ± SD)** | 39.0 ± 1.2 | 39.0 ± 1.1 | 0.60 |  |  |  |
| **Age (years, mean ± SD)** | 31.0 ± 4.6 | 30.5 ± 4.6 | 0.27 |  |  |  |
| **Parity category** |  |  | 0.64 |  |  |  |
| 0 | 61 (41.8%) | 335 (39.2%) |  |  |  |  |
| 1 | 48 (32.9%) | 277 (32.4%) |  |  |  |  |
| 2 | 25 (17.1%) | 141 (16.5%) |  |  |  |  |
| ≥ 3 | 12 (8.2%) | 101 (11.8%) |  |  |  |  |
| **Previous pregnancy loss** | 32 (21.9%) | 180 (21.1%) | 0.82 | 1.04 (0.75-1.45) |  |  |
| **Ethnicity** |  |  | 0.70 |  |  |  |
| Malay | 96 (65.8%) | 525 (61.5%) |  |  |  |  |
| Chinese | 15 (10.3%) | 112 (13.1%) |  |  |  |  |
| Indian | 15 (10.3%) | 101 (11.8%) |  |  |  |  |
| Others | 20 (13.7%) | 116 (13.6%) |  |  |  |  |
| **BMI (kg/m^2^, mean ± SD)** | 29.8 ± 5.2 | 28.7 ± 4.8 | 0.011 |  | 1.00 (0.96-1.04) | 0.95 |
| **Hb < 11 g/dl** | 42 (28.8%) | 170 (19.9%) | 0.015 | 1.45 (1.08-1.93) | 1.44 (0.93-2.24) | 0.11 |
| **Medical & Obstetrics history** |  |  |  |  |  |  |
| **Diabetes in pregnancy** | 47 (32.2%) | 195 (22.8%) | 0.015 | 1.41 (1.08-1.84) | 1.46 (0.95-2.25) | 0.082 |
| **Antiglycemic** | 13 (8.9%) | 57 (6.7%) | 0.33 | 1.33 (0.75-2.38) |  |  |
| **Asthma** | 10 (6.8%) | 43 (5.0%) | 0.37 | 1.36 (0.70-2.65) |  |  |
| **Hypertension in pregnancy** | 10 (6.8%) | 39 (4.6%) | 0.24 | 1.50 (0.77-2.94) |  |  |
| **Group B streptococcus carriage** | 12 (8.2%) | 47 (5.5%) | 0.20 | 1.49 (0.81-2.75) |  |  |
| **Thalassemia trait** | 2 (1.4%) | 8 (0.9%) | 0.63 | 1.46 (0.31-6.82) |  |  |
| **HIV or Hepatitis B infection** | 0 (0.0%) | 9 (1.1%) | 0.21 | * |  |  |
| **Previous Cesarean** | 42 (28.8%) | 123 (14.4%) | < 0.001 | 2.00 (1.48-2.71) | 1.35 (0.85-2.13) | 0.20 |
| **Induction of labor** | 35 (24.0%) | 171 (20.0%) | 0.28 | 1.20 (0.87-1.65) |  |  |
| **Birth weight (kg)** | 3.19 ±0.43 | 3.06 ±0.39 | < 0.001 |  | 1.90 (1.15-3.11) | 0.012 |
| **Cesarean delivery** | 105 (71.9%) | 202 (23.7%) | < 0.001 | 3.04 (2.60-3.56) | 7.27 (4.83-10.95) | <0.001 |

Values are stated as mean ± standard deviation, median [interquartile range] or n (%). Crude analysis by t test for continuous data, Mann-Whitney U test for non-parametric data and Chi Square test for categoric variable. Adjustment made utilizing multivariable binary logistic regression analysis of significant independent variable with adjusted results shown if variable incorporated in the model.
